# Production performance, nutrient use efficiency, and predicted enteric methane emissions in dairy cows under confinement or grazing management system

**DOI:** 10.1093/tas/txac028

**Published:** 2022-02-26

**Authors:** Andre F Brito, Kleves V Almeida, Andre S Oliveira

**Affiliations:** 1 Department of Agriculture, Nutrition, and Food Systems, University of New Hampshire, Durham, NH, 03824, USA; 2 Dairy Cattle Research Laboratory, Universidade Federal de Mato Grosso, Campus Sinop, Sinop, MT, 78557-267, Brazil

**Keywords:** climate change, dairy cow, feed efficiency, greenhouse gas, milk nitrogen efficiency

## Abstract

There has been an intense debate regarding the economic, social, and environmental sustainability of confinement versus grazing dairy systems. Our goal was to conduct a meta-analysis to compare dry matter intake, milk yield and composition, nutrient use efficiency (i.e., feed efficiency, milk N efficiency), and predicted enteric CH_4_ emissions using studies that simultaneously evaluated confinement and grazing. We were able to include in the meta-analysis 8 peer-reviewed articles that met the following selection criteria: (1) publication between 1991 and 2021 in English language, (2) report either SEM or SD, (3) inclusion of at least 1 confinement [total mixed ration or fresh cut herbage fed indoors (i.e., zero-grazing)] and 1 grazing treatment in the same study, and (4) use of markers (internal or external) to estimate herbage dry matter intake. Two unpublished experiments were added to the data set resulting in a total of 10 studies for comparing confinement and grazing. The magnitude of the effect (i.e., effect size) was evaluated using weighted raw mean differences between grazing and confinement systems for a random effect model. Enteric CH_4_ production was predicted as follows: CH_4_ (g/d) = 33.2 (13.54) + 13.6 (0.33) × dry matter intake + 2.43 (0.245) × neutral detergent fiber. Dry matter intake (–9.5%), milk yield (–9.3%), milk fat yield (–5.8%), milk protein yield (–10%), and energy-corrected milk (–12%) all decreased in grazing versus confined dairy cows. In contrast, concentration of milk fat and feed efficiency (energy-corrected milk/dry matter intake) were not affected by management system. Whereas milk protein concentration increased, milk nitrogen (N) efficiency (milk N/N intake) tended to decrease in grazing compared with confinement. Predicted enteric CH_4_ production was 6.1% lower in grazing than confined dairy cows. However, CH_4_ yield (g/kg of dry matter intake) and CH_4_ intensity (g/kg of energy-corrected milk) did not change between confinement and grazing. In conclusion, while production performance decreased in grazing dairy cows, nutrient use efficiency and predicted enteric CH_4_ emissions were relatively similar in both management systems. Results of our meta-analysis should be interpreted with caution due to the small number of studies that met our inclusion criteria leading to a limited number of treatment mean comparisons.

## INTRODUCTION

Pasture-based systems are known to perform multiple ecosystem services including food production, climate regulation, pollination, nutrient cycling, and erosion control (Sala et al., 2017; Tittonell, 2021), as well as use of marginal lands not suitable for tillage and crop production (Wang et al., 2021). Grazing ruminants can also express their natural behaviors while on pasture (Charlton et al., 2011), and previous research revealed that dairy cows were more motivated to go outside to graze than stay indoors and consume a total mixed ration (TMR) offered immediately after the afternoon milking (von Keyserlingk et al., 2017). Another benefit associated with pasture-based dairies is the reduction in production costs, which have been shown to decrease as the proportion of grazed herbage increases in the diet dry matter (DM; Kelly et al., 2020). Furthermore, consumers are willing to pay premiums for pasture-based milk and dairy products due to potential human-health benefits (Benbrook et al., 2018; Stampa et al., 2020; Peira et al., 2020) and the perception that grazing is more environmentally friendly and welfare sounder than confinement (Wong et al., 2010; Bir et al., 2020; Joubran et al., 2021). This opens opportunities to small dairies capitalize on organic certified and grassfed milk markets to remain economically viable (Brito and Silva, 2020; Snider et al., 2021). However, only 10 to 15% of milk produced worldwide comes from grazing operations (Shalloo et al., 2018) and, in Europe and Australia, inclusion of grazed herbage in dairy diets has been declining (Hennessey et al., 2020; Joubran et al., 2021). On the other hand, TMR-based, confinement dairy farms are more prolific not only in the United States (Winsten et al., 2010), but also globally (Joubran et al., 2021) mostly driven by greater milk output compared with grazing dairies (Fontaneli et al., 2005; Winsten et al., 2010; Joubran et al., 2021). Despite a growing interest in the economic and environmental sustainability of confinement and grazing enterprises, we are not aware of any meta-analysis that has compared experiments in which animal production and environmental impact metrics were concurrently measured in both systems.

There is an ongoing interest to better understand differences in nutrient use efficiency and environmental performance of confinement versus grazing dairy systems. However, due to the limited number of studies that had simultaneously investigated the economic, social, and environmental outcomes associated with confinement and pasture-based farms (Tittonell, 2021), a head-to-head comparison between systems is challenging. O’Neill et al. (2011) reported reductions in CH_4_ production (–37%), CH_4_ yield (–11%), and CH_4_ intensity (–13%) in dairy cows grazing perennial ryegrass (*Lolium perenne* L.) herbage compared with those fed TMR. However, these positive responses occurred at expense of DM intake (DMI) and milk yield, which together decreased 27% with feeding the herbage diet (O’Neill et al., 2011). Further evaluations using a larger data set are needed to better understand how diets impact enteric CH_4_ production in dairy cows under confinement or grazing management. We aimed, via a meta-analytical approach, to compare DMI, milk yield and composition, nutrient use efficiency (i.e., feed efficiency, milk nitrogen (N) efficiency), and precited enteric CH_4_ production in studies that simultaneously used confined and grazing dairy cows.

## METHODOLOGY

### Literature Search, Study Eligibility Criteria, and Data Sets

A systematic literature search was conducted using the advanced search webtool of Web of Science (https://www.webofscience.com), Google Scholar (https://scholar.google.com), and Science Direct (https://www.sciencedirect.com). The original search used the key words “grazing” “confinement” “dairy cows” and “methane production” covering the years from 1991 through 2021 in each database. The terms “indoor” and “outdoor” and “milk production” were also used in a second literature search to obtain additional peer-reviewed papers. In the present meta-analysis, the grazing treatment was defined as cows having exclusively access to pasture (i.e., 100% grazed herbage diet) or cows having access to pasture supplemented with partial TMR (pTMR) or conserved forage (i.e., baleage) plus concentrate (Table 1).  Confinement was defined as cows fed TMR or fresh cut herbage (i.e., zero grazing) indoors (Table 1).

**Table 1.** Summary of studies included in the meta-analysis to compare confinement versus grazing dairy systems^1^

Reference	n-cows	DIM^2^	Exp. design^3^	Treatments^4^	Grazed herbage
Civiero et al. (2021)	9	136	3 × 3 LS	(1) TMR, (2) GRAZ + 75% pTMR, (3) GRAZ + 50% pTMR	Pearl millet (*Pennisetum glaucum* ‘Campeiro’)
Soutto et al. (2020)	14	148	RCBD	(1) TMR, (2) GRAZ + CONC	Red oats (*Avena byzantina*)
Fajardo et al. (2015)	41	-^5^	RCBD	(1) TMR, (2) 6 h GRAZ + pTMR, (3) 9 h GRAZ + pTMR	Legume-grass mix [tall fescue (*Festuca arundinacea*), white clover (*Trifolium repens*), and birdsfoot trefoil (*Lotus corniculatus*)]
O’Neill et al. (2011)	48	64	RCBD	(1) TMR, (2) 100% GRAZ	Perennial ryegrass (*Lolium perenne*)
Kaufmann et al. (2011)	14	38	Crossover	(1) Z-GRAZ + CONC, (2) GRAZ + CONC	66% grass with 43% perennial ryegrass (*L. perenne),* 20% herbs with 18% dandelion (*Taraxacum officinale*), and 14% white clover (*T. repens*)
Mohammed et al. (2009)	6	76	3 × 3 LS	(1) Z-GRAZ + CONC, (2) grass silage + CONC, (3) GRAZ + CONC	Perennial ryegrass (*L. perenne*)
Bargo et al. (2002)	45	109	RCBD	(1) TMR, (2) GRAZ + CONC, (3) GRAZ + pTMR	50% smooth bromegrass (*Bromus inermis*), 33% orchardgrass (*Dactylis glomerata*), 7% Kentucky bluegrass (*Poa pratensis*), and 10% weeds and dead herbage
Kolver and Muller (1998)	19	59	CRD	(1) TMR, (2) 100% GRAZ	53% perennial ryegrass (*L. perenne*), 19% white clover (*T. repens*), 21% other grasses including orchardgrass (*D. glomerata*), Kentucky bluegrass (*P. pratensis*), smooth bromegrass (*B. inermis*), and tall fescue (*F. arundinacea*), 3% weeds, and 4% dead herbage
Brito et al. (Study 1)^6^	18	153	RCBD	(1) TMR, (2) GRAZ + legume-grass mix baleage + CONC	90% forage canola (*Brassica napus*), 4.4% grasses, 0.78% legumes, and 4.9% weeds
Brito et al. (Study 2)^6^	20	161	RCBD	(1) TMR, (2) GRAZ + pTMR	81.5% forage canola (*B. napus*), 16% weeds, 2.4% dead herbage

Studies included Holstein (*n* = 5), Holstein-Friesian (*n =* 2), Jersey (*n* = 2), and Holstein × Jersey cross (*n =* 1).

DIM, days in milk.

LS, Latin square; RCBD, randomized complete block design; CRD, completely randomized design.

TMR, total mixed ration, GRAZ, grazing, pTMR, partial total mixed ration, CONC, concentrate, Z-GRAZ, zero-grazing (fresh cut herbage fed in confinement).

Days in milk averaged 24 ± 10 d during herbage DMI measurements in wk 4 and 5 of the study, and milk yield was recorded during wk 0 to 10 in the study.

Unpublished grazing studies conducted at the University of New Hampshire (Durham); diets were formulated to yield a 60:40 forage:concentrate ratio, with forage canola herbage set to replace 30% (Study 1) or 40% (Study 2) of legume-grass mix baleage in the diet dry matter.

The inclusion criteria for selected peer-reviewed papers were: (1) published between 1991 and 2021 (i.e., last 30 years) in English language, (2) report either SEM or SD for variables of interest, (3) inclusion of at least 1 confinement and 1 grazing treatment in the same study, and (4) use of markers (internal or external) to estimate herbage DMI. Our meta-analysis followed the Preferred Reporting Items for Systematic Reviews and Meta-Analyses (PRISMA) guidelines (Moher et al., 2009), with the literature search protocol detailed below. We originally obtained 1,044 publications, with 198 excluded after an initial screening due to duplication of records. The remaining 846 records were screened, and 97 publications were removed because they were defined as systematic reviews, reviews, or meta-analyses. An additional 749 references did not meet the inclusion criterion of simultaneously comparing at least 1 confinement versus 1 grazing treatment in the same study and were excluded from our data set. Twenty-five full-text articles were selected, but further screening resulted in the removal of 17 publications as authors did not report SEM or have not used markers to estimate herbage DMI. Therefore, 8 peer-reviewed papers from the literature search were included in the final data set. Two studies (Brito et al., unpublished) conducted at the University of New Hampshire (Durham) were included to improve the robustness of the data set to detect differences in the variables used to compare confinement versus grazing.

### Calculations

Dry matter intake, milk yield, and concentration and yield on milk components were obtained from treatment means reported in the selected studies (Table 1). Variables that were not reported in tables or text such as feed efficiency and milk N efficiency were calculated. Standard deviation presented herein was obtained from reported SD or computed from SEM multiplied by the square root of experimental units of individual studies. Energy-corrected milk (ECM) yield was calculated according to Orth (1992) as follows: ECM yield = [0.327 × milk yield (kg/d)] + [12.95 × milk fat yield (kg/d)] + [7.2 × milk protein yield (kg/d)]. Feed efficiency was calculated by dividing ECM yield by DMI. When not reported, crude protein intake (kg/d) was calculated by multiplying DMI (kg/d) by the respective treatment crude protein concentration and converted to N intake (g/d) using the 6.25 conversion factor. Milk N was obtained by dividing milk protein by 6.38, with milk N efficiency determined by the division between milk N yield and N intake (reported or calculated) multiplied by 100.

Only 4 studies (2 published and 2 unpublished) included in our data set reported enteric CH_4_ production (O’Neill et al., 2011; Civiero et al., 2021; Brito et al., unpublished). Therefore, we used the intercontinental equation proposed by Niu et al. (2018), which is based on DMI and dietary neutral detergent fiber concentration to predict enteric CH_4_ production for all selected studies including those that measured CH_4_ production. The equation adopted from Niu et al. (2018) was: CH_4_ (g/d) = 33.2 (13.54) + 13.6 (0.33) × DMI + 2.43 (0.245) × neutral detergent fiber. Methane yield was obtained by dividing predicted CH_4_ production (g/d) by measured DMI (kg/d), and CH_4_ intensity by the division between predicted CH_4_ production (g/d) and calculated ECM (kg/d).

### Statistical Analysis

Effect of management system on variable responses (i.e., DMI, N intake, milk yield, ECM yield, milk composition, feed efficiency, milk N efficiency, predicted CH_4_ production, calculated CH_4_ yield, calculated CH_4_ intensity) was evaluated using weighted raw mean differences (WMD) comparing grazing and confinement treatment means (i.e., estimated effect size). The estimated effect size was weighted by the inverse of the variance in the respective studies using the method proposed by DerSimonian and Laird (1986) for a random effect model. Publication bias was assessed using funnel plot asymmetry (Light and Pillemer, 1984) and Egger’s regression method (Egger et al., 1997). The chi-squared (*Q*) test and *I*^*2*^ statistic, which measures the proportion of variation due to heterogeneity (Higgins et al., 2003), were both used to evaluate between-study variability (i.e., heterogeneity of effect size). Heterogeneity values of < 25%, 25 to 50%, and > 50% indicate low, moderate, and high between-study variability, respectively (Higgins et al., 2003). The *metafor* package of R Software (version 1.3.1093; Viechtbauer, 2010; https://cran.rproject.org/web/packages/metafor/metafor.pdf) was used for obtaining WMD, publication bias, *I*^2^ statistics, and forest plot (data not shown). Differences were declared at *P* ≤ 0.05 and tendencies at 0.05 < *P* ≤ 0.10.

## RESULTS AND DISCUSSION

This study was designed to compare production performance, nutrient utilization, and predicted enteric CH_4_ production in dairy cows under confinement or grazing system via a meta-analysis using studies that simultaneously test both treatments. However, we did not aim to oppose both management systems, but rather to fill knowledge gaps while acknowledging the limitations and strengths of confinement and grazing.

Description of the experimental design and treatments from studies used in the meta-analysis is presented in Table 1, and descriptive statistics in Table 2. The same dairy breeds (i.e., Holstein, Holstein-Friesian, Jersey) and crossbred cows (Holstein × Jersey) were used within study (Tables 1 and 2), thus indicating that comparisons between confinement and grazing were not biased by differences in genetic potential. We did not observe publication bias (*P* ≥ 0.08) for milk yield, concentrations of milk fat and protein, and milk protein yield based on funnel plot asymmetry (data not shown) and Egger’s regression asymmetry test (Table 3). In contrast, publication bias was detected for DMI (*P* = 0.03) and milk fat yield (*P* = 0.01) possibly because of the limited number of treatment mean comparisons (n = 14) and associated variation. Heterogeneity values ranged from low (*I*^2^ = 22%; *P* = 0.21; milk fat concentration) to high (*I*^2^ = 95.3%; *P* < 0.01; DMI) as shown in Table 3. Specifically, heterogeneity was considered high (>50%; mean *I*^2^ = 88.3%) for all variables analyzed except milk fat concentration (Table 3), thus indicating large between-study variability.

**Table 2.** Descriptive statistics of studies used in the meta-analysis to compare confinement versus grazing dairy systems^1^

Item^2^	*n-*study	*n*-treatment	Mean	± SD	Minimum	Maximum
Confinement						
Body weight, kg	10	-^3^	567	61.8	460	660
Days in milk	10	-^3^	94.0	46.7	24.0	161
DMI, kg/d	10	11	21.9	3.45	15.6	26.7
Milk yield, kg/d	10	11	30.2	9.01	16.1	44.1
Milk fat, %	10	11	4.06	0.66	3.30	5.32
Milk fat, kg/d	10	11	1.15	0.23	0.58	1.56
Milk protein, %	10	11	3.30	0.27	2.80	3.88
Milk protein, kg/d	10	11	0.98	0.26	0.47	1.30
ECM, kg/d	10	11	31.6	7.42	15.9	43.2
Feed efficiency, kg/kg	10	11	1.43	0.21	1.02	1.92
N intake, g/d	10	11	583	104	342	720
Milk N efficiency, %	10	11	25.2	4.27	16.6	30.6
CH_4_ production, g/d	10	11	420	39.3	369	473
CH_4_ yield, g/kg of DMI	10	11	20.7	3.05	17.6	26.9
CH_4_ intensity, g/kg of ECM	10	11	15.1	3.87	10.1	23.3
Grazing						
Body weight, kg	10	-^3^	561	69.8	433	660
Days in milk	10	-^3^	94.0	46.7	24.0	161
DMI, kg/d	10	13	19.9	2.40	14.3	25.2
Milk yield, kg/d	10	13	27.3	6.19	19.6	42.4
Milk fat, %	10	13	4.10	0.61	3.13	5.41
Milk fat, kg/d	10	13	1.09	0.19	0.83	1.59
Milk protein, %	10	13	3.40	0.31	2.82	4.03
Milk protein, kg/d	10	13	0.88	0.17	0.64	1.32
ECM, kg/d	10	13	29.4	5.51	21.5	43.5
Feed efficiency, kg/kg	10	13	1.48	0.22	1.23	2.03
N intake, g/d	10	13	620	112	467	768
Milk N efficiency, %	10	13	23.0	6.79	16.5	37.6
CH_4_ production, g/d	10	13	403	32.8	340	460
CH_4_ yield, g/kg of DMI	10	13	20.5	3.03	14.1	26.9
CH_4_ intensity, g/kg of ECM	10	13	15.5	3.40	9.70	20.9

Studies included Holstein (*n* = 5), Holstein-Friesian (*n =* 2), Jersey (*n* = 2), and Holstein × Jersey cross (*n =* 1); confinement was defined as a management system with cows fed total mixed ration, fresh cut herbage (zero-grazing), or grass silage indoors, and grazing as a management system with cows having access to pasture and consuming herbage as the sole dietary ingredient, herbage supplemented with partial total mixed ration, or herbage supplemented with baleage plus concentrate.

DMI (dry matter intake); ECM (energy-corrected milk) yield = [0.327 × milk yield (kg/d)] + [12.95 × milk fat yield (kg/d)] + [7.2 × milk protein yield (kg/d)] (Orth, 1992); feed efficiency = ECM yield/DMI; milk N efficiency = (milk N/N intake) × 100; predicted CH_4_ production (g/d) = 33.2 (13.54) + 13.6 (0.33) × DMI + 2.43 (0.245) × neutral detergent fiber (Niu et al., 2018); CH_4_ yield was obtained by dividing predicted CH_4_ production by measured DMI; CH_4_ intensity was obtained by dividing predicted CH_4_ production by calculated ECM yield.

Studies did not report days in milk and body weight by treatment.

**Table 3.** Effect of confinement (CONF) or grazing dairy management system on dry matter intake (DMI) and milk yield and composition^1^

Item	CONF mean(SD)	n^2^	WMD (95% CI)^3^		Heterogeneity^4^		Funnel test^5^
			Random effect	*P*-value	*P*-value	*I* ^2^(%)	*P-*value
DMI, kg/d	21.9 (3.45)	14	−2.09 (−3.49, −0.69)	<0.01	<0.01	95.3	0.03
Milk yield, kg/d	30.2 (9.01)	14	−2.82 (−5.11, −0.51)	<0.01	<0.01	92.1	0.08
Milk fat, %	4.1 (0.66)	14	0.04 (−0.05, 0.13)	0.38	0.21	22.0	0.84
Milk protein, %	3.3 (0.27)	14	0.08 (0.00, 0.16)	0.03	<0.01	71.6	0.83
Milk fat, kg/d	1.2 (0.23)	14	−0.07 (−0.14, −0.00)	0.05	<0.01	88.8	0.01
Milk protein, kg/d	1.0 (0.26)	14	−0.10 (−0.18, −0.01)	<0.01	<0.01	93.8	0.17

Confinement was defined as a management system with cows fed total mixed ration, fresh cut herbage (zero-grazing), or grass silage indoors, and grazing as a management system with cows having access to pasture and consuming herbage as the sole dietary ingredient, herbage supplemented with partial total mixed ration, or herbage supplemented with baleage plus concentrate.

n, number of treatment mean comparisons between confinement and grazing.

WMD, weighted raw mean differences between confinement and grazing (i.e., size effect) using the method proposed by DerSimonian and Laird (1986) for a random effect model; CI, confidence interval.

*P*-value for χ2 (*Q*) test of heterogeneity; *I*^2^ = proportion of total variation of size effect estimates that is due to heterogeneity (i.e., between-study variability).

Egger’s regression asymmetry test (Egger et al., 1997).

Effect of management system on DMI, milk yield, and concentration and yield of milk fat and protein assessed via WMD between confinement and grazing is presented in Table 3. Dry matter intake decreased (*P* < 0.01) by 9.5% in grazing dairy cows compared with those under confinement management. Grazing cows spend more time searching and selecting food than confined dairy cows (Agnew and Yan, 2000), which can limit the amount of herbage consumed leading to less total DMI (Reis and Combs, 2000). It should be also noted that grazing cows are generally more exposed to heat and heat stress conditions resulting in less grazing activity and decreased herbage DMI.

Milk yield was 9.3% lower (*P* < 0.01; Table 3) in grazing versus confined dairy cows likely in response to a 9.5% drop in DMI leading to decreased energy intake. In 3 studies used in the data set (i.e., Fajardo et al., 2015; Soutto et al., 2020; Civiero et al., 2021), grazing cows received less concentrate than those in confinement, while in 2 other experiments (i.e., Kolver and Mullen, 1998; O’Neill et al., 2011), herbage was not supplemented with concentrate (100% grazing; Table 1). Therefore, decreased or no concentrate supplementation also contributed to the milk yield reduction in grazing dairy cows (Table 3). Furthermore, increased energy requirement due to grazing activity (i.e., energy spent to select and consume herbage) and walking back and forth from pasture to the milking parlor, shifts dietary energy away from milk synthesis to maintenance in pasture-based dairy cows (Agnew and Yan, 2000; NRC, 2001). Bargo et al. (2002) estimated, using equations reported in the NRC (2001), that compared with confined cows fed TMR, maintenance requirements increased by 5.4 and 2.4 Mcal/d in grazing dairy cows supplemented with concentrate or pTMR, respectively. According to Bargo et al. (2002), increased maintenance requirement accounted for 88 and 61% of the differences in milk yield between cows offered TMR versus herbage supplemented with concentrate or pTMR, respectively.

Management system did not affect the concentration of milk fat (*P* = 0.38) as shown in Table 3. In contrast, concentration of milk protein increased (*P* = 0.03) by 2.4%, whereas yields of milk fat (*P* = 0.05) and milk protein (*P* < 0.01) decreased by 5.8 and 10%, respectively, between grazing versus confined dairy cows (Table 3). Increased milk protein concentration can be explained by a dilution effect caused by increased milk volume. Decreased production of milk fat and protein followed the reduction in milk yield (–9.3%), with all linked to lowered DMI (–9.5%) in pasture-based diets. Overall, grazing decreased yields of milk and milk fat and protein, and these production losses may not be offset by less feed costs often associated with pasture-based diets as American dairy farmers receive premiums for shipping more fat and protein. Hardie et al. (2014) demonstrated via a cluster analysis using 69 organic-certified dairy farms from Wisconsin that dairies feeding the least amount of concentrate and relying heavily on pasture had lower milk rolling herd average (mean = 3,632 kg/cow per year) and income over feed costs ($5.76/lactating cow per d) than those with greatest concentrate and least reliance on grazed herbage (mean = 6,878 kg/cow per year of milk rolling herd average and $10.2/lactating cow per d of income over feed costs). However, organic grassfed milk markets can potentially counteract production losses due to additional premiums paid to farmers (Benbrook et al., 2018; Brito and Silva, 2020; Snider et al., 2021)

**Figure 1.**
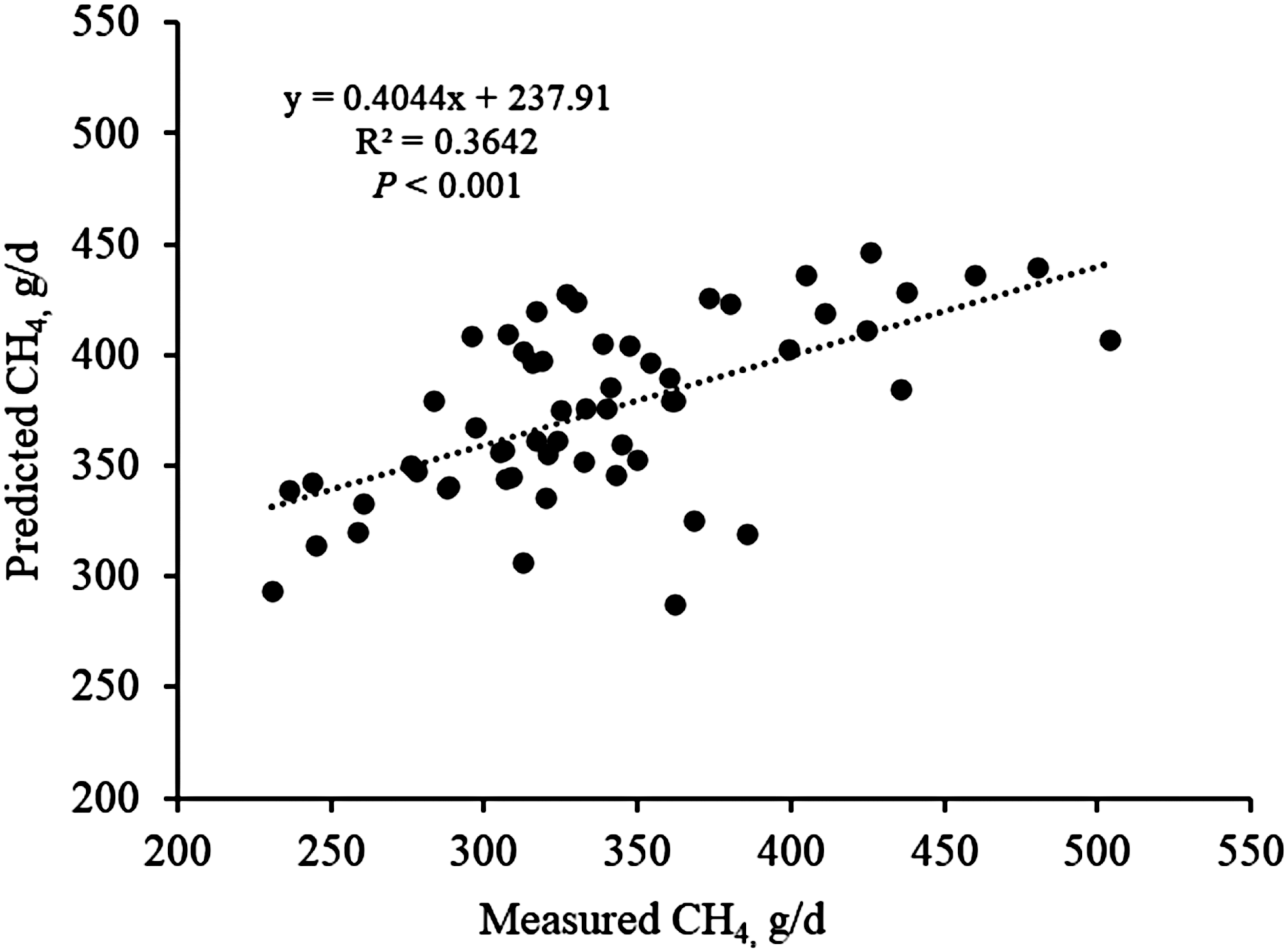
Relationship between measured and predicted CH_4_ production in grazing dairy cows. Individual CH_4_ production observations (*n* = 58) were obtained from lactating dairy cows grazing cool season legume-grass mix herbage (Antaya et al., 2019) or forage canola herbage (Brito et al., unpublished). Diets from the 2 unpublished studies were formulated to yield a 60:40 forage:concentrate ratio, with forage canola herbage set to replace 30% (Study 1) or 40% (Study 2) of legume-grass mix baleage in the diet dry matter. Enteric CH_4_ production was predicted using one of the intercontinental equations published by Niu et al. (2018): CH_4_ production (g/d) = 33.2 (13.54) + 13.6 (0.33) × dry matter intake + 2.43 (0.245) × neutral detergent fiber.

Effect of management system on ECM yield, feed efficiency, milk N efficiency, and predicted enteric CH_4_ production evaluated through WMD between confinement and grazing is presented in Table 4. Response variables shown in Table 4 were all calculated or predicted to standardize comparisons between management systems and to obtain additional data such as enteric CH_4_ production, which was reported in only 4 out of 10 studies [O’Neill et al., 2011; Civiero et al., 2021; Brito et al., unpublished (2 experiments)]. Therefore, the intercontinental equation [CH_4_ (g/d) = 33.2 (13.54) + 13.6 (0.33) × DMI + 2.43 (0.245) × neutral detergent fiber] published by Niu et al. (2018) was used to predict CH_4_ production. This equation was developed using a refined data set containing 2,566 individual observations of enteric CH_4_ production obtained from 42 studies conducted in Europe (*n* = 1,423), 45 in the United States (*n* = 1,084), and 1 study from Australia (*n* = 59). Holstein was the predominant dairy breed, contributing with 68% (*n* = 1,732) of the total individual observations followed by Ayrshire (19%; *n* = 497), Brown Swiss, Simmental, and crossbred dairy cattle 10% (*n* = 249), and Jersey (3%; *n* = 88). It should be noted that none of the experiments conducted in Europe or United States used grazing dairy cows, and only 1 pasture-based study (2.3%; *n* = 59) was included in the final data set, suggesting that equations reported by Niu et al. (2018) could be more accurate to predict enteric CH_4_ production from confined than grazing cows. We used 58 individual observations of enteric CH_4_ production from 3 grazing studies in which diets were formulated to contain (DM basis) 30% or 40% of forage canola herbage (*Brassica napus* L.; Brito et al., unpublished; Table 1), or 48% of cool season legume-grass mix herbage (Antaya et al., 2019) to assess the relationship between measured and predicted CH_4_ production via regression (Figure 1). Despite the limited number of observation (*n* = 58), there was a moderate relationship between measured and predicted CH_4_ production (R^2^ = 0.36; *P* < 0.001) indicating that the equation of Niu et al. (2018), which is based on DMI and dietary neutral detergent fiber concentration, appears to be reliable to predict CH_4_ production in grazing dairy cows consuming (actual intake) up to 51% of herbage (% of diet DM). It is also important to note that the prediction equation used herein had one the greatest concordance correlation coefficient (i.e., 0.75) and smallest mean absolute error (i.e., 48.5 g/d) indicating that CH_4_ production can be reasonably predicted (Niu et al., 2018).

**Table 4.** Effect of confinement (CONF) or grazing dairy management system on energy-corrected milk yield, nutrient use efficiency, and predicted enteric CH_4_ production^1^

Item^2^	CONF mean (SD)	*n* ^3^	WMD (95% CI)^4^	
			Random effect	*P*-value
ECM, kg/d	31.6 (7.42)	14	–3.88	<0.01
Feed efficiency, kg/kg	1.43 (0.21)	14	0.09	0.20
N intake, g/d	583 (104)	14	30.3	0.52
Milk N efficiency, %	25.2 (4.27)	14	−2.78	0.09
CH_4_ production, g/d	420 (39.3)	14	−25.7	<0.01
CH_4_ yield, g/kg DMI	20.7 (3.05)	14	0.18	0.86
CH_4_ intensity, g/kg ECM	15.1 (3.87)	14	1.31	0.23

Confinement was defined as a management system with cows fed total mixed ration, fresh cut herbage (zero-grazing), or grass silage indoors, and grazing as a management system with cows having access to pasture and consuming herbage as the sole dietary ingredient, herbage supplemented with partial total mixed ration, or herbage supplemented with baleage plus concentrate.

ECM (energy-corrected milk) yield = [0.327 × milk yield (kg/d)] + [12.95 × milk fat yield (kg/d)] + [7.2 × milk protein yield (kg/d)] (Orth, 1992); feed efficiency = ECM yield/dry matter intake; milk N efficiency = (milk N/N intake) × 100; predicted CH_4_ production (g/d) = 33.2 (13.54) + 13.6 (0.33) × dry matter intake + 2.43 (0.245) × neutral detergent fiber (Niu et al., 2018); CH_4_ yield was obtained by dividing predicted CH_4_ production by measured DMI; CH_4_ intensity was obtained by dividing predicted CH_4_ production by calculated ECM yield.

n, number of treatment mean comparisons between confinement and grazing.

WMD, weighted raw mean differences between confinement and grazing (i.e., size effect) using the method proposed by DerSimonian and Laird (1986) for a random effect model; CI, confidence interval.

Energy-corrected milk yield decreased (*P* < 0.01) by 12% in grazing versus confined dairy cows (Table 4), which is in line with reduced yields of milk and milk fat and protein (Table 3). Contrarily, management system did not affect feed efficiency (*P* = 0.20). Nitrogen intake was not impacted by management system (*P* = 0.52), but milk N efficiency tended (*P* = 0.09) to decrease with grazing (Table 4), which may be associated with greater concentration of soluble crude protein in herbage than TMR (Bargo et al., 2002). In general, improved feed efficiency and milk N efficiency indicate that cows are more efficient in partitioning nutrients for production of milk and milk components than waste including enteric CH_4_ and nitrogenous compounds such as urinary urea N. However, the lack of management system effect on feed efficiency, and only a trend for improving milk N efficiency with confinement implies similar nutrient use efficiency between confined cows and those with access to pasture.

Predicted enteric CH_4_ production was 6.1% lower (*P* < 0.01)  in grazing than confined dairy cows (Table 4), thus in line with reduced DMI (Table 3). In fact, it is well known that DMI is positively correlated with enteric CH_4_ production in lactating dairy cows (Hristov et al., 2013, 2018). Neither CH_4_ yield (g/kg of DMI) nor CH_4_ intensity (g/kg of ECM) changed (*P* ≥ 0.23) in response management system (Table 4). We detected a more pronounced reduction in CH_4_ production  (i.e., –19%) in grazing (mean = 368 g/d) versus confinement (mean = 453 g/d; data not shown) when using data from selected studies (*n* = 4; O’Neill et al., 2011; Civiero et al., 2021; Brito et al., unpublished) whereby enteric CH_4_ production was directly measured. Furthermore, CH_4_ yield (–7.2%) and CH_4_ intensity (–6.2%) were both lower in cows under grazing than confinement management in these 4 studies (data not shown). In 3 out of 4 studies from this smaller data set, cows grazed high quality herbage in the form of perennial ryegrass (mean = 24.1% crude protein; mean = 46.5% neutral detergent fiber; O’Neill et al., 2011) or forage canola (mean = 24.5% crude protein; mean = 16.1% ash-free neutral detergent fiber; Brito et al., unpublished), which likely contributed to the larger reduction in enteric CH_4_ emissions compared with the complete data set (*n* = 10 studies). Forage canola also contains glucosinolates that have been shown to be negatively correlated with CH_4_ production in continuous culture (Dillard et al., 2018). Overall, the enteric CH_4_ production data reported in Table 4 should be interpreted cautiously because we used an equation to predict enteric CH_4_ production as discussed previously.

Enteric CH_4_ accounts for approximately 27% of total CH_4_ emissions in the United States (EPA, 2019). Even though the atmospheric half-life of CH_4_ (~10 years) is much shorter than that of other greenhouse gases such as N_2_O (~110 years) and CO_2_ (~1,000 years), its global warming potential is about 28 times greater compared with that of CO_2_ (Lashof et al., 1990; IPCC, 2013). In addition to its effects on global warming, enteric CH_4_ represents energy losses ranging from 2.7 to 9.8% of gross energy intake in lactating dairy cows (Niu et al., 2018). Therefore, dietary and management strategies to mitigate enteric CH_4_ emissions in ruminants can improve both the carbon footprint of dairy farms and milk yield of dairy cows. Our meta-analysis revealed only a small difference in predicted CH_4_ production between confinement and grazing systems, and no changes in CH_4_ yield and CH_4_ intensity (Table 4). However, a fair comparison and evaluation of dairy management systems should also consider greenhouse gas emissions from crop production, transportation, and manure management, as well as ecosystem services provided by grazing dairies (Fredeen et al., 2013 ; Tittonell, 2021), which was beyond the scope of our study. Nevertheless, results from studies that have compared the carbon footprint of grazing and confinement dairy systems are not consistent. For instance, whereas some studies reported reduced whole-farm greenhouse gas emissions in grazing versus confinement (Flysjö et al., 2011; O’Brien et al., 2014), others showed increased emissions with grazing management (Capper et al., 2009; Léis et al., 2015). In contrast, Aguirre-Villegas et al. (2017) reported comparable whole-farm greenhouse gas emissions across different grazing and confinement scenarios using Wisconsin dairies in their modeling simulations.

## CONCLUSIONS

Our meta-analysis provided a snapshot of production performance, nutrient use efficiency, and predicted enteric CH_4_ emissions of confinement versus grazing dairy systems using studies that simultaneously compared these 2 management approaches. We showed that yields of milk, milk fat and protein, and ECM were all lower (ranging from –5.8 to –12%) in grazing than confinement, with these responses mostly driven by decreased DMI (–9.5%) in cows with access to pasture. Feed efficiency did not change, and milk N efficiency tended to decrease with grazing, thus indicating similar nutrient utilization between both systems. Predicted CH_4_ production decreased by 6.1% in grazing dairy cows due to reduced DMI. However, CH_4_ yield (g/kg of DMI) and CH_4_ intensity (g/kg of ECM) were not affected by management system. In general, results of our meta-analysis should be interpreted cautiously due to the limited number of studies (*n* = 10) and associated treatment mean comparisons (*n* = 14) that met inclusion criteria. We also used a published equation based on DMI and dietary neutral detergent fiber concentration to predict CH_4_ production because only 4 studies used in the data set directly measured CH_4_. Whole-farm greenhouse gas emissions and ecosystems services provided by grazing should be considered in future assessments of confinement and pasture-based dairy systems.
